# Gallstones: new insights into an old story

**DOI:** 10.12688/f1000research.8874.1

**Published:** 2016-07-26

**Authors:** Evan Tiderington, Sum P. Lee, Cynthia W. Ko

**Affiliations:** 1 Division of Gastroenterology, University of Washington, Seattle, WA, USA

**Keywords:** gallstones, biliary colic, gallstone pancreatitis, acute cholecystitis, cholecystectomy, cholesterol stone disease, management of gallstone disease

## Abstract

Gallstones, particularly cholesterol gallstones, are common in Western populations and may cause symptoms such as biliary colic or complications such as acute cholecystitis or gallstone pancreatitis. Recent studies have allowed for a better understanding of the risk of symptoms or complications in patients with gallstones. In addition, newer data suggest an association of gallstones with overall mortality, cardiovascular disease, gastrointestinal cancers, and non-alcoholic fatty liver disease. Knowledge of appropriate indications and timing of cholecystectomy, particularly for mild biliary pancreatitis, has gradually accumulated. Lastly, there are exciting possibilities for novel agents to treat or prevent cholesterol stone disease. This review covers new advances in our understanding of the natural history, clinical associations, and management of gallstone disease.

## Introduction

Gallstones are crystallized deposits containing cholesterol and/or bilirubin that form most commonly in the gallbladder and have been found in autopsy studies from Egyptian and Chinese mummies. In Western countries, gallstones are composed primarily of cholesterol. In developing nations and Asia, mixed or pigment stones, which contain a higher proportion of bilirubin, are seen more frequently than in Western countries. Gallstone disease is defined as the presence of gallstones accompanied by symptoms attributable to their presence (biliary colic) or complications such as acute cholecystitis, acute cholangitis, and gallstone pancreatitis. Gallstones are very common, occurring in up to 20% of adults in Europe and the United States, with significant variations in prevalence worldwide based on both genetic and environmental factors ^1, 2^. For example, the prevalence of gallstones is close to 50% in American Indians and over 35% in Chileans with Amerindian background ^3, 4^. Associated with the high burden of gallstones in these populations is a high risk for gallbladder cancer ^5, 6^. Currently, cholecystectomy is the gold standard for the treatment of gallstone disease, and over 700,000 cholecystectomies are performed annually in the United States.

Although gallstones have been known to cause symptoms and complications for centuries, our understanding of the natural history and clinical associations between gallstones and related diseases continues to evolve, as does our understanding of the risks and benefits of modern surgical and medical interventions. In this article, we discuss recent literature about the natural history of gallstones and potential advances in the medical and surgical management of gallstone-related disease. We do not focus on recent advances in gallstone pathophysiology or genetics, which have recently been extensively reviewed by others ^7– 9^.

## Natural history

Although gallstones are common in the general population, only a minority of patients with gallstones develop symptoms or complications. The presence of symptoms or complications is important in deciding appropriate therapy, but to date few studies have estimated overall and relative risks of developing symptoms in initially asymptomatic patients ^10– 15^. In addition, the studies that have estimated rates of complications have generally been smaller and have reported widely ranging risk of symptoms or complications.

A recent population-based study by Shabanzadeh *et al*. attempts to fill this knowledge gap ^16^. This study analyzed data from three randomly selected groups participating in an international study of cardiovascular risk factors in the general population. Overall, 664 patients with sonographically detected gallstones were followed for a mean of 17.4 years. The primary outcome was hospital admission for gallstone-related diagnoses or treatments, with further analyses specifically of complicated or uncomplicated events. Almost 20% of subjects had gallstone-related events during the follow-up period. Female sex, the presence of multiple stones, and largest stone size greater than 10 mm were associated with higher risk of uncomplicated and complicated events, while age was inversely associated.

The authors developed a clinical prediction score based on these variables to estimate the absolute and relative risks of gallstone-related events. For example, the absolute risk of symptomatic or complicated gallstone disease was 3.2 per 1000 person-years for a male with a solitary small stone but increased to 23.5 per 1000 person-years for a female with multiple stones and at least one stone larger than 10 mm (hazard ratio [HR] 11.05, 95% confidence interval [CI] 3.76–32.44 compared with the lowest-risk male). The findings that stone size and multiple stones are associated with risk are consistent with some, but not all, previous studies, but the sampling frame of the different studies varies ^14, 17– 19^. Thus, these results require further confirmation, and the clinical prediction score needs validation in other cohorts. However, these results may help clinicians in discussing risk of future complications and appropriate treatment options in patients with asymptomatic gallstones. Patients deemed to be at low risk for future adverse outcomes may be counseled to avoid treatment, while patients with high risk for morbidity might be counseled to consider earlier cholecystectomy.

## Association of gallstones with overall mortality, cardiovascular disease, cancer, and non-alcoholic fatty liver disease

While the majority of patients with asymptomatic gallstones do not progress to have gallstone-related symptoms or complications, growing evidence suggests that gallstones may be associated with increased overall mortality and with other medical problems including cardiovascular disease, non-alcoholic fatty liver disease (NAFLD), and cancer. In this section, we review recent population-based studies examining these associations.

### Overall mortality

There is emerging evidence to suggest that gallstone disease is either an important marker of an increased risk for medical comorbidities or an important etiologic factor in the development of medical comorbidities. For example, gallstones may be associated with risk for overall mortality, cardiovascular disease, cancer, and NAFLD. A recent analysis of the population-based third US National Health and Nutrition Examination Survey (NHANES III) data by Ruhl and Everhart demonstrated an association between gallstone disease and increased mortality ^20^. The analysis included participants in NHANES III with linkage to the National Death Index to ascertain subjects’ vital statuses. Gallstone disease was defined as the presence of gallstones on study ultrasonography or evidence of prior cholecystectomy. Subjects with gallstone disease had a 30% greater all-cause mortality over the follow-up period (adjusted HR 1.3, 95% CI 1.1–1.5), including a 40% greater mortality from cardiovascular disease (adjusted HR 1.4, 95% CI 1.2–1.7), and a 30% greater mortality from cancer (adjusted HR 1.3, 95% CI 0.98–1.8). These associations held even after adjusting for expected confounders including age, gender, race/ethnicity, alcohol and tobacco consumption, body mass index, waist circumference, and diabetes. In this study, stones were identified sonographically and the biochemical composition of the stones was not determined. As this was a US-based study, the majority of gallstones were probably cholesterol stones, and this association is likely valid in this patient population. However, it is unclear whether mixed or pigment stones are also associated with increased overall mortality.

### Cardiovascular disease

Recent studies have further examined a possible association of gallstones with cardiovascular risk. Lv *et al*. performed a population-based prospective cohort study in China of over 500,000 adults aged 30 to 79 years without baseline heart disease or prior cerebrovascular events ^21^. Subjects were followed for the development of ischemic heart disease through local disease and death registries. The presence of gallstones was determined through self-report and, therefore, the composition of the gallstones was not known. Over a median follow-up of 7.2 years, the risk of developing incident ischemic heart disease was greater in people who self-reported a history of gallstone disease. This association held even after adjusting for age, gender, body mass index, physical activity level, diabetes, hypertension, smoking status, and a family history of heart attack, with an adjusted HR of 1.23 (95% CI 1.17–1.28). The association between gallstones and cardiovascular disease was stronger for women (adjusted HR 1.27, 95% CI 1.20–1.34) compared to men (adjusted HR 1.11, 95% CI 1.02–1.22).

Wirth *et al*. conducted another population-based, prospective cohort study to investigate this potential association ^22^. This study enrolled over 46,000 subjects aged 35 to 65 without cardiovascular disease or diabetes at baseline. Diagnosis of gallstones was ascertained by self-report, and gallstone composition was not documented. At baseline, 10.4% of participants reported the presence of gallstones, with 66.5% of those subjects also reporting a history of cholecystectomy. Over an average follow-up of 8.2 years, subjects with reported gallstones had an increased risk of cardiovascular disease (HR 1.24, 95% CI 1.02–1.50).

Both of these studies relied on participant self-reporting of gallstone disease and cholecystectomy and were not able to assess the risk of incident ischemic heart disease in those with asymptomatic and undiagnosed gallstones. Therefore, these results may underestimate the risk of cardiovascular disease in this patient population. Although gallstone composition was not determined in any of these studies, these results provide support for an association between gallstone disease as a whole and an increased risk of incident cardiovascular disease.

### Cancer

The relationship between gallstones and cancer was studied further in a population-based case-control study by Nogueira *et al*. ^23^. Cancer cases were obtained from the Surveillance, Epidemiology, and End Results (SEER) database. Controls were randomly selected Medicare recipients who were cancer free at the time of selection and were frequency-matched to cases by calendar year of selection, age, and gender. Gallstone and cholecystectomy exposure were defined by the presence of specific ICD-9 claim codes at least 12 months prior to cancer diagnosis or control selection. As might be expected, gallbladder cancer was strongly associated with the presence of gallstones (odds ratio [OR] 3.13, 95% CI 2.82–3.48) but inversely associated with past cholecystectomy (OR 0.07, 95% CI 0.04–1.15). There were statistically significant associations with gastric cancer overall and non-cardia gastric cancer, both for gallstones (OR for non-cardia gastric cancer 1.21, 95% CI 1.11–1.32) and for prior cholecystectomy (OR for non-cardia gastric cancer 1.26, 95% CI 1.13–1.40). Gallstones or cholecystectomy were also significantly associated with hepatocellular carcinoma, cholangiocarcinoma, pancreatic cancers, and cancers of the ampulla of Vater, with stronger associations for gallstone exposure compared to cholecystectomy exposure. Interestingly, although prior studies have suggested an association between gallstones and colorectal cancer ^24– 28^, gallstones and cholecystectomy were not statistically significantly associated with cancers of the proximal colon in this study. However, there was a borderline inverse association with cancers of the distal colon (OR 0.94, 95% CI 0.89–1.00 for gallstones) and a significant inverse association with cancers of the rectum (OR 0.83, 95% CI 0.78–0.89 for gallstones, and OR 0.85, 95% CI 0.78–0.92 for cholecystectomy). As this study was performed in a US population, it is likely that the majority of gallstones studied were cholesterol stones, and we again do not know if these associations are also true for mixed or pigment stones. In addition, *Helicobacter pylori* and other *Helicobacter* species have been found in association with gallstones ^29, 30^, which may account for some of the observed associations with gastric cancer. *Helicobacter* status was not examined in this study, so we cannot determine if this is a confounding factor. Nevertheless, these results add to our understanding of the overall mortality risk associated with cholelithiasis ^20^ by providing more granular data on potential associations of gallstones with specific gastrointestinal cancer sites.

### Non-alcoholic fatty liver disease

There is also a growing appreciation of an association between gallstones and NAFLD. In an analysis of the population-based NHANES III data, the overall prevalence of NAFLD was 20% and was doubled in patients with gallstone disease compared to those without gallstone disease (37.5% vs. 17.7%, p<0.001) ^31^. In adjusted analyses, the presence of gallstones was associated with a 56% increase in the odds of NAFLD (95% CI 27–91%). Interestingly, the presence of NAFLD was more strongly associated with prior cholecystectomy than with ultrasound-diagnosed gallstones. In a cross-sectional study of 482 patients, Koller *et al*. found that the prevalence of cholelithiasis was nearly doubled in patients with markers of NAFLD (45.5% vs. 27.5%, p<0.0001), while cholelithiasis, type 2 diabetes mellitus, and body mass index greater than 25 kg/m ^2^ were independent risk factors for NAFLD ^32^. Similar to the other population-based studies described above, the investigators did not determine the chemical composition of stones in this study. Nevertheless, this study suggests that cholelithiasis itself may have metabolic consequences that predispose to NAFLD and that the association between gallstones and NAFLD is not solely due to shared risk factors.

### Clinical mechanisms and implications

Although the recent literature suggests broad associations between gallstone disease and a range of clinical outcomes, the design of these studies does not allow the determination of a causal effect. In addition, the population-based studies were not able to determine the chemical composition of stones, and it is unclear whether the reported associations pertain to cholesterol stones, pigment stones, or both. Gallstones may not themselves be causative in the development of cardiovascular disease or cancer, and the associations noted may reflect shared underlying risk factors. For example, gallstone prevalence is known to be associated with obesity, diabetes, and insulin resistance ^33– 36^, and the association between gallstones and increased cardiovascular disease incidence may simply reflect the presence of these shared risk factors. In addition, gallstones are associated with alterations in signaling in the farnesoid X receptor (FXR) and liver X receptor (LXR) pathways, and these abnormalities may also play a role in metabolic regulation ^37– 40^. Similar considerations apply for the association between gallstones and NAFLD. Nevertheless, the study by Ruhl and Everhart ^20^ found an association between gallstone disease and mortality even after adjustment for potential confounders including waist circumference and diabetes, suggesting that gallstone disease as a whole may still represent an independent risk factor for adverse clinical outcomes.

The mechanisms of association between gallstones and digestive system cancers also remain speculative. Gallstones have been demonstrated to be associated with pancreatic, gallbladder, and biliary tract cancers ^41– 43^. Biliary tract cancers may arise due to the chronic inflammation and biliary stasis which is common in patients with cholelithiasis. The association between gallstones and colorectal cancer remains controversial, with studies variously showing positive, negative, or no association ^24– 28^. In addition, cholecystectomy is known to alter bile acid composition and increase the enterohepatic circulation of bile, with increased concentrations of secondary bile acids throughout the gastrointestinal tract which may be carcinogenic and pro-inflammatory.

Further study is needed to determine if gallstones are a marker for increased mortality, cardiovascular disease, or NAFLD independent of other risk factors such as obesity, diabetes, and insulin resistance. However, clinicians may consider counseling patients with gallstones more broadly on measures to control weight and also use the diagnosis of gallstones as an impetus to more aggressively control metabolic risk factors.

## Management

The management of gallstones and related conditions is complex, and cholecystectomy is generally considered to be the gold standard for the management of symptomatic or complicated disease. Nevertheless, the timing of cholecystectomy continues to be a matter of debate ^44– 46^. Medical management of cholesterol stone disease with ursodeoxycholic acid is not uniformly successful, and not all patients are candidates for this therapy ^9^. Recent studies, reviewed here, have shed light on the appropriate timing of cholecystectomy for patients with mild acute biliary pancreatitis and have raised the possibility for a much-needed novel medical treatment for cholesterol gallstones. More comprehensive reviews of the treatment of gallstones are available elsewhere ^9, 44, 45, 47^.

### Surgical management

Patients with acute cholecystitis or acute pancreatitis frequently do not undergo cholecystectomy during their initial hospitalization but instead undergo interval cholecystectomy at a later date because of concern for complications with earlier surgery. However, there may be a higher risk of recurrent biliary events in patients who undergo interval surgery.

In a randomized, hospital-based trial, Da Costa *et al.* compared same-admission and delayed cholecystectomy for patients with mild acute biliary pancreatitis ^48^. In total, 266 patients were randomized to undergo same-admission surgery or cholecystectomy 25 to 30 days later. Seventeen percent of the delayed surgery group were later readmitted for biliary complications, compared to 5% of the same-day surgery group (relative risk [RR] 0.28, 95% CI 0.12–0.66). Recurrent pancreatitis occurred in 9% of the delayed surgery group compared to 2% in the same-admission surgery group (RR 0.27, 95% CI 0.08–0.92). There was no significant difference in rates of conversion to open cholecystectomy, perceived difficulty of surgery, or surgical complications. Thus, early surgical management in mild biliary pancreatitis appears to be safe and does not increase complications over the common practice of delayed surgery. These findings cannot necessarily be generalized to patients with moderate-to-severe pancreatitis or to patients with other biliary complications such as acute cholecystitis. However, they may provide greater motivation to current practitioners not to delay cholecystectomy for mild biliary pancreatitis. Further studies to examine early vs. delayed cholecystectomy for patients with acute cholecystitis or with moderate-to-severe biliary pancreatitis are also needed.

### Medical management

Although surgical management of gallstones is generally safe and effective, medical options for the prevention and treatment of gallstones continue to evolve as well. An important pathophysiologic factor in the development of cholesterol stones is the hepatic hypersecretion of cholesterol. Ursodeoxycholic acid lowers biliary cholesterol supersaturation and can be effective in dissolving cholesterol stones in select patients ^7^. However, rates of recurrent stone formation are high, and this therapy has generally fallen out of favor ^7^. Therefore, new therapies that can both prevent and treat cholesterol stones are needed. Recent studies have suggested a role for ezetimibe, which inhibits intestinal absorption of cholesterol, in the prevention and treatment of cholesterol gallstones. In murine studies, ezetimibe significantly reduced hepatic secretion of cholesterol and prevented estrogen-induced formation of gallstones ^49^. This effect was also seen in patients with documented gallstones, in whom ezetimibe significantly reduced biliary cholesterol secretion and slowed cholesterol crystallization ^50^. Although larger-scale human studies have not been performed, ezetimibe may be an alternative for the prevention and treatment of cholesterol stones in high-risk patients.

## Summary

Although gallstones have plagued humans since ancient times, our understanding of the natural history, clinical implications, and management of gallstones continues to evolve. We are slowly gaining an understanding of the natural history of gallstones, which will allow us to better understand when to intervene medically or surgically. For example, we may counsel patients predicted to be at low risk for future adverse outcomes to avoid treatment, while patients with high risk for morbidity might be counseled to consider earlier intervention. Gallstones, in particular cholesterol stones, may portend an increased risk of mortality, cardiovascular disease, and cancer, and their presence may indicate a need for providers to more aggressively identify and manage metabolic and cardiovascular risk factors or to be more aggressive in evaluating symptoms suggestive of digestive tract cancers. Finally, our understanding of the proper medical and surgical management is steadily increasing, and we are gradually developing clinical protocols to guide appropriate timing of cholecystectomy. Nevertheless, further studies are urgently needed to define appropriate indications for surgery and to examine novel agents for medical prevention and therapy.
